# New-onset nontuberculous mycobacterial pulmonary disease in bronchiectasis: tracking the clinical and radiographic changes

**DOI:** 10.1186/s12890-020-01331-3

**Published:** 2020-11-10

**Authors:** Nakwon Kwak, Jong Hyuk Lee, Hyung-Jun Kim, Sung A. Kim, Jae-Joon Yim

**Affiliations:** 1 Division of Pulmonary and Critical Care Medicine, Department of Internal Medicine, Seoul National University College of Medicine, 101 Daehak-Ro, Jongno-Gu, Seoul, 110-744 South Korea; 2 Department of Radiology, Seoul National University College of Medicine, Seoul, South Korea

**Keywords:** Mycobacterium, Incidence, Infection

## Abstract

**Background:**

The close association between bronchiectasis and nontuberculous mycobacterial pulmonary disease (NTM-PD) is well-known. However, the clinical impact of subsequent new-onset NTM-PD in bronchiectasis patients has not been elucidated. The aim of this study is to investigate the clinical courses and radiographic changes of patients with bronchiectasis in whom NTM-PD subsequently developed.

**Methods:**

A total of 221 patients with bronchiectasis who had participated in a non-NTM bronchiectasis cohort between July 1st 2011 and August 31st 2019 at Seoul National University Hospital were included in this study. The data of patients in whom NTM-PD developed during this observation period were analyzed; specifically, changes in the Bronchiectasis Severity Index (BSI) and lesions on computerized tomography (CT) scan of the chest arising during the observation period.

**Results:**

During the observation period, NTM was isolated from 35 patients. A total of 31 patients (14.0%) satisfied the diagnostic criteria of NTM-PD. The median time from enrollment in the cohort to the development of subsequent NTM-PD was 37 months (Interquartile range [IQR], 18–78 months). *Mycobacterium avium* complex was the most common pathogen (80.6%). Twelve patients underwent antibiotic treatment for NTM-PD with a median interval of 20 months (IQR, 13–30) from the time of NTM-PD diagnosis. When NTM-PD developed, the severity and extent of bronchiectasis, cellular bronchiolitis, and the extent of nodules worsened on CT scans, while BSI did not change.

**Conclusions:**

NTM-PD can develop in previously negative bronchiectasis patients. It is associated with worsening radiographic lesions. Active screening of non-NTM bronchiectasis patients for new-onset NTM infection should be considered, especially if radiographic findings worsen. The BSI is not a reliable predictor of new-onset NTM-PD.

**Trial registration:**

This study was performed at Seoul National University Hospital (NCT01616745).

**Supplementary Information:**

The online version contains supplementary material available at 10.1186/s12890-020-01331-3.

## Background

Bronchiectasis is a chronic respiratory disease characterized by airway dilatation leading to impaired mucociliary clearance and persistent airway inflammation resulting in further airway damage [1]. It is a heterogeneous disease in terms of its etiology, co-existing morbidities, and prognosis [1, 2]. Although bronchiectasis has been a neglected orphan disease, its incidence has been gradually increasing, especially in older age groups [3, 4]. Bronchiectasis is associated with increased mortality [4].

Nontuberculous mycobacterial pulmonary disease (NTM-PD) can cause bronchiectasis directly. When it occurs in the presence of bronchiectasis, it can cause disease progression [1]. According to the U.S. Bronchiectasis Research Registry, about 60% of patients with bronchiectasis have had a previous history of NTM-PD or had NTM cultured from sputum [5]. NTM infection accounted for the etiology in 9.4% of patients with bronchiectasis in the U.S. population [6]. NTM-infected patients were diagnosed with bronchiectasis at a later age and had more dilated airways than those without NTM infection [5].

In patients with bronchiectasis from other causes, the anatomically altered bronchi are vulnerable to NTM infection [1, 7]. The clinical impact of the development of NTM-PD in patients with underlying bronchiectasis has not been elucidated, especially in patients without cystic fibrosis [8]. In this study, we investigated the clinical course and radiographic changes in bronchiectasis patients with new-onset NTM-PD.

## Methods

### Participants

Our hospital has a prospective cohort of patients with bronchiectasis. All patients included in this analysis provided written informed consents to participate in the protocol, and the Institutional Review Board of Seoul National University Hospital approved the study (IRB No.1806–034-949). This study was conducted in accordance with the amended Declaration of Helsinki. A total of 221 patients with bronchiectasis who had participated in a non-NTM bronchiectasis cohort between July 1st 2011 and August 31st 2019 at Seoul National University Hospital were included (NCT01616745). Some of these patients had been included in previous studies [9–12]. The diagnosis of bronchiectasis was based on the radiographic features in thin-section computerized tomography (CT) scans as follows: 1) bronchial dilatation with respect to the accompanying pulmonary artery, 2) lack of tapering of the bronchial lumen, and 3) identification of bronchi within 1 cm of the pleural surface [13]. The absence of NTM infection was confirmed when negative growth of acid-fast bacilli (AFB) was proven at least two times more than 1 month apart after entry into the non-NTM bronchiectasis cohort.

Once diagnosed with bronchiectasis, the patients visited the clinics every 8–24 weeks according to their condition. AFB staining and mycobacterial culture of sputum were requested every year if patients could expectorate sputum and additional AFB staining and mycobacterial cultures could be requested at the discretion of the physician on duty based on symptomatic and radiographic changes. Pulmonary function tests and thin section CT of the chest were performed every 2 years.

When NTM was isolated from the sputum ≥2 times or bronchoalveolar lavage and the clinical and radiographic findings were compatible with NTM-PD according the criteria suggested by American Thoracic Society (ATS)/Infectious Diseases Society of America (IDSA) [14], a diagnosis of NTM-PD was made. These patients were included for the final analysis.

### Bronchiectasis severity index (BSI)

The BSI score, which was developed to predict the risk of mortality, hospital admission, and exacerbation in patients with bronchiectasis [15], was measured at the time of initial enrollment of non-NTM bronchiectasis cohort as well as at the time of NTM-PD diagnosis. BSI score was calculated using the following variables: Age, body mass index (BMI), forced expiratory volume in 1 s (FEV1) % predicted, hospital admission during the last 2 years, exacerbations during the last 1 year (a deterioration in three or more of cough, sputum volume/consistency, sputum purulence, breathlessness/exercise tolerance, fatigue/malaise and hemoptysis for at least 48 h) [16], Medical Research Council (MRC) dyspnea score, colonization with *Pseudomonas* and/or other organisms, the number of involved lobes, or cystic bronchiectasis.

### CT scoring

Chest CT scans were scored in terms of bronchiectasis (9 points maximum, with up to 3 points each assigned to degree of severity, extent, and mucus plugging), cellular bronchiolitis (6 points maximum, with up to 3 points each assigned to degree of severity and extent), cavity formation (9 points maximum, with up to 3 points each assigned to diameter, wall thickness, and extent). Nodules and consolidation were each scored with up to 3 points (absent, mild, moderate-to-severe) and the extent of involved segments (absent, 1–5 segments, 6–9 segments, or > 9 segments), respectively [17]. All CT scans were reviewed by a board-certified radiologist (LJH).

### Statistical analysis

Data are described as median values with interquartile ranges (IQRs) or proportions. For the categorical variables, the Chi-squared test and Fisher’s exact test were used. The Wilcoxon signed-rank test was performed to compare continuous variables measured at the time of enrollment and at the time of NTM-PD diagnosis. All analyses were performed using commercial software (SPSS version 23.0®, SPSS Inc., Chicago IL, USA).

## Results

**Fig. 1:**
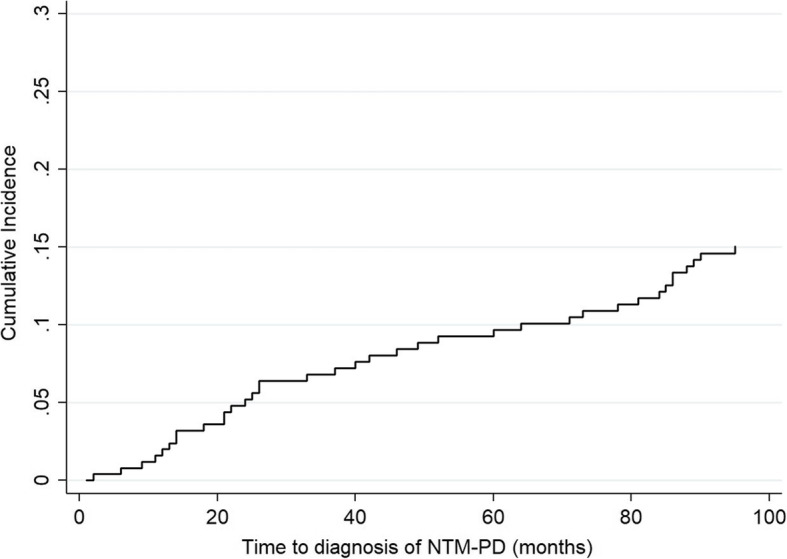
Cumulative incidence curve of NTM-PD in non-NTM bronchiectasis

The median follow-up duration for the 221 patients was 55 months (IQR 38–70). During this time, NTM was isolated from 35 (15.8%) patients (30 patients from sputum and 5 patients from bronchoalveolar lavage. Among them, 31 (14.0%) patients satisfied ATS/IDSA diagnostic criteria for NTM-PD. The patients with new-onset NTM-PD did not differ from the NTM-negative patients in terms of age, BMI, smoking history, previous history of tuberculosis, respiratory symptoms, initial FEV1% predicted, bacterial colonization, radiographic findings and BSI. However, three or more exacerbations of bronchiectasis (*P* = 0.001) and hospital admission (*P* = 0.007) were more frequent in patients with new-onset NTM-PD (Table 1). The median interval from enrollment into the cohort to NTM-PD development was 37 months (IQR 18–78 months) and the incidence rate for the development of NTM-PD was 32.3 per 1000 person-years (95% CI 22.7–45.9). The Kaplan-Meier plot of cumulative incidence of NTM-PD is illustrated in Fig. 1.

**Table 1:** Baseline characteristics of patients of 31 patients with subsequent NTM-PD and 190 patient without subsequent NTM-PD

	Patients with subsequent NTM-PD (*n* = 31)	Patients without subsequent NTM-PD (*n* = 190)	*P* value
Age, years, median [IQR]	62 (50–65)	61 (54–68)	0.098
Female, n (%)	19 (61.3)	67 (64.7)	0.692
Body mass index, kg/m^2^ median [IQR]	21.4 (19.4–23.7)	22.0 (19.8–24.2)	0.419
Former or current smoker, n (%)	10 (32.3)	36 (19.0)	0.099
Previous history of tuberculosis, n (%)	12 (38.7)	68 (35.8)	0.841
Chronic obstructive pulmonary disease, n (%)	2 (6.5)	20 (10.5)	0.747
Asthma, n (%)	2 (6.5)	2 (1.1)	0.095
Respiratory symptom, n (%)			
Cough	15 (48.4)	82 (43.2)	0.697
Sputum	24 (77.4)	160 (84.2)	0.435
Hemoptysis	11 (35.5)	42 (22.1)	0.116
Initial forced expiratory volume in 1 s (%), median [IQR]	96 (72–103)	88 (72–104)	0.519
Exacerbation during the last 1 year			0.001
0	22 (71.0)	128 (67.4)	
1–2	2 (6.4)	52 (27.4)	
3 or more	7 (22.6)	10 (5.2)	
Hospital admission during the last 2 years, n (%)	9 (29.0)	19 (10.0)	0.007
Pseudomonas colonization, n (%)	0	4 (2.1)	> 0.999
Colonization with other organisms, n (%)	2 (6.5)	17 (9.0)	0.646
≥3 lobes involved, n (%)	17 (54.8)	104 (54.7)	> 0.999
Presence of cavity, n (%)	2 (6.5)	19 (10.0)	0.746
Bronchiectasis severity score, median [IQR]	4 [3–5]	4 [2, 7]	0.963

*NTM-PD* nontuberculous mycobacterial pulmonary disease, *IQR* inter-quartile range

The most frequently encountered species was *Mycobacterium avium* (17, 54.8%), followed by *M. intracellulare* (8, 25.8%). *M. abscessus* subspecies *abscessus* was isolated in two patients. The other four patients were infected with two different species (*M. avium*/*M. intracellulare* in three patients and *M. avium*/*M. abscessus* subspecies *massiliense* in one patient). None of these patients were treated with a macrolide during the follow-up period, and the *M. avium* isolates from one patient showed macrolide resistance.

A total of 12 (25.8%) of 31 patients received antibiotic treatment after a median of 47 months (IQR 16–103) from enrollment in the cohort and 20 months (IQR 13–30) from the diagnosis of NTM-PD because of radiographic aggravation (5 patients), symptomatic aggravation (5 patients) or both (2 patients) (Table 2). The mycobacterial species at the time of treatment was the same as that at the initial diagnosis in these patients and 7 patients achieved microbiological cure. BMI did not change from entry into the cohort (21.8 [IQR 19.4–23.8]) to the diagnosis of NTM-PD (21.8, [IQR 19.9–23.9]) (*P* = .948). The median FEV1% predicted was 93% (IQR 70–102) and 96% (IQR 76–107) at the time of non-NTM bronchiectasis cohort entry and NTM-PD diagnosis, respectively (*P* = .06) (Table 2).

**Table 2:** Clinical features and outcomes of 31 patients after the diagnosis of NTM-PD

Time from the diagnosis of bronchiectasis to the diagnosis of NTM-PD, months, median [IQR]	37 [18, 78]
NTM species, n (%)	
*Mycobacterium avium* complex	25 (80.6%)
*M. avium*	17 (54.8)
*M. intracellulare*	8 (25.8)
*Mycobacterium abscessus*	
*M. abscessus*	2 (6.5)
Mixed infection	
*M. avium/ M. intracellulare*	3 (9.7)
*M. avium/ M. massiliense*	1 (3.2)
Treatment initiation, n (%)	12 (25.8)
Time to treatment initiation from the diagnosis of NTM-PD	20 (13–30)

*NTM-PD* nontuberculous mycobacterial pulmonary disease, *IQR* inter-quartile range;

The BSI score at entry into the non-NTM bronchiectasis cohort was a median of 4 (IQR 2–7). It did not change at the time of NTM-PD diagnosis, with a median of 4 (IQR 3–6) (Table 3). The mean CT score increased from 7.6 (standard deviation [SD], 2.9) on entry into the non-NTM bronchiectasis cohort to 9.1 (SD 2.4) at the time of NTM-PD diagnosis (*P* = .002), while 12 of 31 patients in whom NTM-PD was subsequently diagnosed did not develop radiographic aggravation until diagnosis of NTM-PD. Specifically, severity (*P* = .035) and mucus plugging (*P* = .033) in bronchiectasis, severity (*P* = .007) and extent (*P* = .039) of cellular bronchiolitis, and the extent of nodule distribution (*P* = .034) increased significantly, while scores for cavity formation and consolidation were not changed (Table 4). The detailed scoring results of each patient are provided in Supplementary Table 1.

**Table 3:** Changes in bronchiectasis severity index of 31 patients between the time of entry into the non-NTM bronchiectasis cohort and the time at the diagnosis of NTM-PD

*n* = 31	Entry of non-NTM BE cohort	Diagnosis of NTM-PD	*P*-value
Age, years			0.126
< 50	7 (22.6)	2 (6.5)	
50–69	22 (71.0)	24 (77.4)	
70–79	2 (6.5)	5 (16.1)	
< 80	0	0	
Body mass index, kg/m^2^			0.782
< 18.5	4 (12.9)	4 (12.9)	
18.5–25	23 (74.2)	21 (67.7)	
26–29	4 (12.9)	6 (19.4)	
30 or more	0	0	
FEV1, % predicted			0.570
> 80	21 (67.7)	24 (77.4)	
50–80	10 (32.3)	7 (22.6)	
30–49	0	0	
< 30	0	0	
Hospital admission during the last 2 years			0.554
No	22 (71.0)	25 (80.9)	
Yes	9 (29.0)	6 (19.4)	
Exacerbation during the last 1 year			0.317
0	22 (71.0)	19 (61.3)	
1–2	2 (6.4)	6 (19.4)	
3 or more	7 (22.6)	6 (19.4)	
MRC dyspnea score			> 0.999
1–3	31 (100.0)	31 (100.0)	
4	0	0	
5	0	0	
Pseudomonas colonization			> 0.999
No	31 (100.0)	31 (100.0)	
Yes	0	0	
Colonization with other organisms			> 0.999
No	29 (93.5)	29 (93.5)	
Yes	2 (6.5)	2 (6.5)	
3 or more lobes involved or cystic bronchiectasis			0.434
No	14 (45.2)	10 (31.4)	
Yes	17 (54.8)	21 (67.7)	
Bronchiectasis severity score			0.491
Mild	21 (67.7)	17 (54.8)	
Moderate	6 (19.4)	10 (32.3)	
Severe	4 (12.9)	4 (12.9)	
Total score, median [IQR]	4 [2, 7]	4 [3, 6]	0.903

*BE* bronchiectasis, *NTM-PD* nontuberculous mycobacterial pulmonary disease, *IQR* inter-quartile range

**Table 4:** Changes in CT score of 31 patients between the time of entry into the non-NTM bronchiectasis cohort and the time at the diagnosis of NTM-PD

Mean (SD)	Entry of non-NTM BE cohort	Diagnosis of NTM-PD	*P*-value
Bronchiectasis	3.3 (1.7)	4.0 (1.5)	0.010
Severity	1.5 (0.8)	1.7 (0.7)	0.035
Extent	1.3 (0.6)	1.5 (0.6)	0.059
Mucus plugging	0.6 (0.7)	0.8 (0.8)	0.033
Cellular bronchiolitis	3.5 (1.5)	4.1 (1.1)	0.005
Severity	2.0 (0.8)	2.3 (0.6)	0.007
Extent	1.5 (0.8)	1.8 (0.7)	0.039
Cavity	0.3 (1.3)	0.3 (1.3)	> 0.999
Diameter	0.1 (0.6)	0.1 (0.6)	> 0.999
Wall thickness	0.1 (0.5)	0.1 (0.5)	> 0.999
Extent	0.1 (0.3)	0.1 (0.3)	> 0.999
Nodules	0.3 (0.5)	0.5 (0.5)	0.034
Consolidation	0.2 (0.4)	0.2 (0.4)	> 0.999
Total score	7.6 (2.9)	9.1 (2.4)	0.002

*NTM-PD* nontuberculous mycobacterial pulmonary disease, *SD* standard devation

## Discussion

For this study, we traced 221 patients participating in a non-NTM bronchiectasis cohort. During a median follow-up duration of 55 months, NTM-PD developed in 31 (14.0%) after a median follow-up duration of 37 months. Diagnosis of NTM-PD was accompanied by worsening radiographic findings, while BSI did not change significantly.

Bronchiectasis and NTM-PD are closely linked. A recent meta-analysis reported the overall prevalence of NTM pulmonary infection in patients with bronchiectasis is 9.3% [18]. In this study, the prevalence of NTM-PD in patients with bronchiectasis was 14.0%. The higher prevalence of NTM-PD in South Korea than in other areas, including the United States and Europe, might explain the relative abundance of NTM-PD in this cohort [19–21].

Though the correlation between bronchiectasis and NTM-PD is well-known, the causation is not fully established [22]. NTM infection causes bronchiectasis and it was regarded as an etiology of bronchiectasis in 9.4–18.0% of patients in two studies [6, 23]. Conversely, the presence of bronchiectasis may predispose patients to acquire NTM, which may adhere to the damaged respiratory mucosal surface using fibronectin attachment protein [24]. Individuals with bronchiectasis are 50–75 times more likely to have NTM infection than those without bronchiectasis [3]. While the close linkage between these two entities has been recognized, the research on the impact of NTM infection has mainly focused on bronchiectasis with cystic fibrosis [25, 26].

In our study, 31 out of 221 patients with non-NTM bronchiectasis were subsequently diagnosed with NTM-PD over a median observation time of 37 months. Development of NTM-PD did not affect the BSI score. Clinical parameters included in the BSI score such as BMI, FEV1, number of hospital admissions, or the dyspnea score did not change. However, the radiographic severity worsened in terms of bronchiectasis, cellular bronchiolitis, and nodules. These results suggest that radiographic deterioration in patients with bronchiectasis could be a clue to the development of NTM-PD.

The current guidelines recommend mycobacterial cultures in patients with bronchiectasis only if initiation of long-term macrolide use is planned [27]. Given that subsequent development of NTM-PD (14.0%) was not rare in non-NTM bronchiectasis, that it was accompanied by radiographic worsening, and that treatment for NTM-PD was required in one-third of patients in our study, active screening for NTM infection among bronchiectasis patients is warranted [27]. In addition, considering that the symptoms and signs of bronchiectasis and NTM-PD overlap [8, 27], and that BSI score did not increase with development of subsequent NTM-PD in our study, periodic screening for NTM infection in bronchiectasis patients, regardless of planning macrolide therapy or symptomatic aggravation, should be performed.

## Conclusions

In conclusion, NTM-PD developing in previously NTM-negative bronchiectasis patients is accompanied by worsening radiographic findings. Active screening for NTM infection should be considered for all bronchiectasis patients, especially if radiographic lesions worsen.

## Supplementary Information

**Additional file 1:** **Supplementary Table.** Detailed CT scoring of 31 patients at the time of entry into the non-NTM bronchiectasis cohort and at the time of diagnosis of NTM-PD

## Data Availability

The dataset used are available from the corresponding author on reasonable request.

## Abbreviations

AFB: Acid-fast bacilli
ATS/IDSA: American Thoracic Society/Infectious Diseases Society of America
BMI: Body mass index
BSI: Bronchiectasis Severity Index
CT: Computerized tomography
FEV1: Forced expiratory volume in 1 s
IQR: Interquartile range
MRC: Medical Research Council
NTM-PD: Nontuberculous mycobacterial pulmonary disease
