# Supplementary material for: New-onset nontuberculous mycobacterial pulmonary disease in bronchiectasis: tracking the clinical and radiographic changes
Source: BMC Pulm Med. 2020 Nov 10;20:293. doi: 10.1186/s12890-020-01331-3 (PMC7653824; doi:10.1186/s12890-020-01331-3)
Supplement: Supplementary file 1 — Additional file 1: Supplementary Table. Detailed CT scoring of 31 patients at the time of entry into the non-NTM bronchiectasis cohort and at the time of diagnosis of NTM-PD [file 12890_2020_1331_MOESM1_ESM.docx]

Supplementary Table. Detailed CT scoring of 31 patients at the time of entry into the non-NTM bronchiectasis cohort and at the time of diagnosis of NTM-PD

| ID | At the time of entry into the non-NTM bronchiectasis cohort | | | | | | | | | | | At the time of NTM-PD diagnosis | | | | | | | | | | |
| --- | --- | --- | --- | --- | --- | --- | --- | --- | --- | --- | --- | --- | --- | --- | --- | --- | --- | --- | --- | --- | --- | --- |
|  | Bronchiectasis | | | Cellular bronchiolitis | | Cavity | | | Nodules | Consolidation | Total score | Bronchiectasis | | | Cellular bronchiolitis | | Cavity | | | Nodules | Consolidation | Total score |
|  | Severity | Extent | Mucus plugging | Severity | Extent | Diameter | Thickness | Extent |  |  |  | Severity | Extent | Mucus plugging | Severity | Extent | Diameter | Thickness | Extent |  |  |  |
| 1 | 1 | 1 | 0 | 1 | 1 | 0 | 0 | 0 | 0 | 0 | 4 | 1 | 1 | 0 | 2 | 3 | 0 | 0 | 0 | 1 | 0 | 8 |
| 2 | 0 | 0 | 0 | 0 | 0 | 0 | 0 | 0 | 0 | 0 | 0 | 1 | 1 | 0 | 1 | 2 | 0 | 0 | 0 | 1 | 0 | 6 |
| 3 | 1 | 1 | 0 | 3 | 3 | 0 | 0 | 0 | 1 | 1 | 10 | 1 | 1 | 0 | 3 | 1 | 0 | 0 | 0 | 1 | 0 | 7 |
| 4 | 3 | 3 | 1 | 3 | 3 | 0 | 0 | 0 | 1 | 0 | 14 | 3 | 3 | 2 | 3 | 3 | 0 | 0 | 0 | 1 | 1 | 16 |
| 5 | 2 | 2 | 2 | 2 | 2 | 0 | 0 | 0 | 0 | 0 | 10 | 2 | 2 | 2 | 2 | 1 | 0 | 0 | 0 | 0 | 0 | 9 |
| 6 | 1 | 2 | 0 | 2 | 2 | 0 | 0 | 0 | 0 | 0 | 7 | 2 | 2 | 1 | 2 | 2 | 0 | 0 | 0 | 0 | 0 | 9 |
| 7 | 1 | 1 | 1 | 2 | 1 | 0 | 0 | 0 | 0 | 0 | 6 | 2 | 1 | 1 | 3 | 1 | 0 | 0 | 0 | 0 | 1 | 9 |
| 8 | 2 | 1 | 1 | 1 | 1 | 0 | 0 | 0 | 0 | 0 | 6 | 2 | 1 | 1 | 2 | 1 | 0 | 0 | 0 | 0 | 0 | 7 |
| 9 | 1 | 1 | 0 | 2 | 1 | 0 | 0 | 0 | 0 | 0 | 5 | 2 | 1 | 0 | 2 | 2 | 0 | 0 | 0 | 0 | 0 | 7 |
| 10 | 1 | 1 | 0 | 2 | 1 | 0 | 0 | 0 | 1 | 0 | 6 | 1 | 1 | 0 | 2 | 1 | 0 | 0 | 0 | 1 | 0 | 6 |
| 11 | 2 | 2 | 1 | 2 | 1 | 1 | 2 | 1 | 0 | 0 | 12 | 2 | 2 | 0 | 2 | 1 | 0 | 0 | 0 | 1 | 0 | 8 |
| 12 | 1 | 1 | 1 | 2 | 1 | 0 | 0 | 0 | 0 | 1 | 7 | 2 | 2 | 1 | 3 | 1 | 0 | 0 | 0 | 1 | 1 | 11 |
| 13 | 1 | 1 | 0 | 3 | 1 | 0 | 0 | 0 | 0 | 0 | 6 | 1 | 1 | 1 | 3 | 2 | 0 | 0 | 0 | 0 | 0 | 8 |
| 14 | 1 | 1 | 1 | 2 | 1 | 0 | 0 | 0 | 0 | 0 | 6 | 1 | 1 | 1 | 2 | 2 | 0 | 0 | 0 | 1 | 0 | 8 |
| 15 | 3 | 2 | 0 | 1 | 1 | 0 | 0 | 0 | 0 | 0 | 7 | 3 | 2 | 2 | 2 | 2 | 0 | 0 | 0 | 0 | 0 | 11 |
| 16 | 1 | 2 | 0 | 2 | 1 | 3 | 2 | 1 | 0 | 0 | 12 | 1 | 2 | 0 | 2 | 1 | 3 | 2 | 1 | 0 | 0 | 12 |
| 17 | 2 | 1 | 0 | 2 | 1 | 0 | 0 | 0 | 0 | 0 | 6 | 2 | 1 | 0 | 2 | 1 | 0 | 0 | 0 | 0 | 0 | 6 |
| 18 | 1 | 1 | 0 | 0 | 0 | 0 | 0 | 0 | 1 | 0 | 3 | 2 | 2 | 0 | 1 | 1 | 0 | 0 | 0 | 0 | 0 | 6 |
| 19 | 3 | 2 | 1 | 2 | 1 | 0 | 0 | 0 | 1 | 0 | 10 | 2 | 1 | 0 | 2 | 2 | 0 | 0 | 0 | 1 | 1 | 9 |
| 20 | 3 | 1 | 1 | 2 | 1 | 0 | 0 | 0 | 0 | 1 | 9 | 3 | 1 | 1 | 2 | 1 | 0 | 0 | 0 | 1 | 0 | 9 |
| 21 | 2 | 2 | 1 | 1 | 2 | 0 | 0 | 0 | 1 | 0 | 9 | 2 | 2 | 2 | 3 | 2 | 0 | 0 | 0 | 1 | 0 | 12 |
| 22 | 2 | 2 | 1 | 2 | 2 | 0 | 0 | 0 | 1 | 0 | 10 | 1 | 2 | 2 | 2 | 2 | 0 | 0 | 0 | 1 | 0 | 10 |
| 23 | 2 | 1 | 0 | 2 | 2 | 0 | 0 | 0 | 0 | 1 | 8 | 2 | 1 | 0 | 2 | 2 | 0 | 0 | 0 | 0 | 0 | 7 |
| 24 | 1 | 1 | 1 | 3 | 1 | 0 | 0 | 0 | 0 | 0 | 7 | 1 | 1 | 1 | 3 | 1 | 1 | 2 | 1 | 0 | 0 | 11 |
| 25 | 1 | 1 | 0 | 2 | 1 | 0 | 0 | 0 | 0 | 0 | 5 | 2 | 1 | 1 | 2 | 2 | 0 | 0 | 0 | 0 | 0 | 8 |
| 26 | 1 | 1 | 0 | 3 | 3 | 0 | 0 | 0 | 1 | 0 | 9 | 1 | 2 | 1 | 3 | 3 | 0 | 0 | 0 | 1 | 0 | 11 |
| 27 | 1 | 1 | 0 | 2 | 1 | 0 | 0 | 0 | 1 | 0 | 6 | 1 | 1 | 0 | 2 | 1 | 0 | 0 | 0 | 1 | 0 | 6 |
| 28 | 1 | 2 | 3 | 3 | 2 | 0 | 0 | 0 | 1 | 0 | 12 | 1 | 2 | 3 | 3 | 3 | 0 | 0 | 0 | 1 | 0 | 13 |
| 29 | 2 | 1 | 1 | 3 | 2 | 0 | 0 | 0 | 0 | 0 | 9 | 2 | 1 | 1 | 3 | 2 | 0 | 0 | 0 | 0 | 0 | 9 |
| 30 | 1 | 1 | 0 | 3 | 2 | 0 | 0 | 0 | 0 | 1 | 8 | 2 | 2 | 1 | 3 | 3 | 0 | 0 | 0 | 0 | 1 | 12 |
| 31 | 0 | 0 | 1 | 2 | 3 | 0 | 0 | 0 | 0 | 1 | 7 | 1 | 1 | 1 | 2 | 3 | 0 | 0 | 0 | 1 | 1 | 10 |
